# Investigation of Cerebellar Abiotrophy (CA), Lavender Foal Syndrome (LFS), and Severe Combined Immunodeficiency (SCID) Variants in a Cohort of Three MENA Region Horse Breeds

**DOI:** 10.3390/genes12121893

**Published:** 2021-11-26

**Authors:** Abdelhanine Ayad, Saria Almarzook, Omar Besseboua, Sofiane Aissanou, Katarzyna Piórkowska, Adrianna D. Musiał, Monika Stefaniuk-Szmukier, Katarzyna Ropka-Molik

**Affiliations:** 1 Department of Environment and Biological Sciences, Faculty of Nature and Life Sciences, University of Bejaia, Bejaia 06000, Algeria; abdelhanine.ayad@univ-bejaia.dz (A.A.); sofiane.aissanou@univ-bejaia.dz (S.A.); 2 Faculty of Applied Sciences, University of Applied Sciences Europe, 10963 Berlin, Germany; saria.almarzook@ue-germany.de; 3 Department of Agronomic and Biotechnological Sciences, Faculty of Nature and Life Sciences, University H. Benbouali, Chlef 02000, Algeria; besseboua.omar@gmail.com; 4 Department of Animal Molecular Biology, National Research Institute of Animal Production, Krakowska 1, 32-083 Balice, Poland; katarzyna.piorkowska@iz.edu.pl (K.P.); adrianna.musial@iz.edu.pl (A.D.M.); m.k.stefaniuk@gmail.com (M.S.-S.)

**Keywords:** genetic disorders, Arabian horses, Barb horses, Arab-Barb horses, CA, LFS, SCID, molecular biology

## Abstract

Genetic disorders in horses are mostly fatal or usually cause significant economic losses for breeders and owners. Here we studied a total of 177 Arabian, Barb and Arab-Barb horses from the Middle East and North Africa (MENA) using Sanger Sequencing and PCR-ACRS (polymerase chain reaction—artificially created restriction site) approaches to examine the genetic disorders in the studied horse breeds. We identified the genetic variations related to Cerebellar Abiotrophy (CA), Severe Combined Immunodeficiency (SCID) occurrence, and the studied population was free of the mutant allele determined Lavender Foal Syndrome (LFS). Overall, presented data showed that 15 of the studied horses are carriers of two genetic disorders; the investigated horse population showed that five Arabian horses were heterozygous for the CA-associated SNP (rs397160943). The SCID-deletion TCTCA within *PRKDC* was detected in ten horses (nine Arabian horses and one Arab-Barb horse). This investigation shows the importance of testing these breeds for genetic disorders to avoid further spread of deleterious variants

## 1. Introduction

The earliest evidence of horse domestication is described in Botai culture 5500 years before present in central Asian steppe. This process may have taken place in different places at different times [1] such as Anatolia, the Pontic Caspian Steppes, and Iberian Peninsula regions [2]. Recent genomic research indicates a cultural and geographic context in which the modern horse lineage emerged [3]. The spread of the horses from Mesopotamia to North-Eastern Arabia, later reaching South Arabia in what is known today as the MENA region (the Middle East and North Africa) is based on archaeological evidence of horse remains appearing on archaeological sites in the Levant during early historical periods [4].

**Figure 1:**
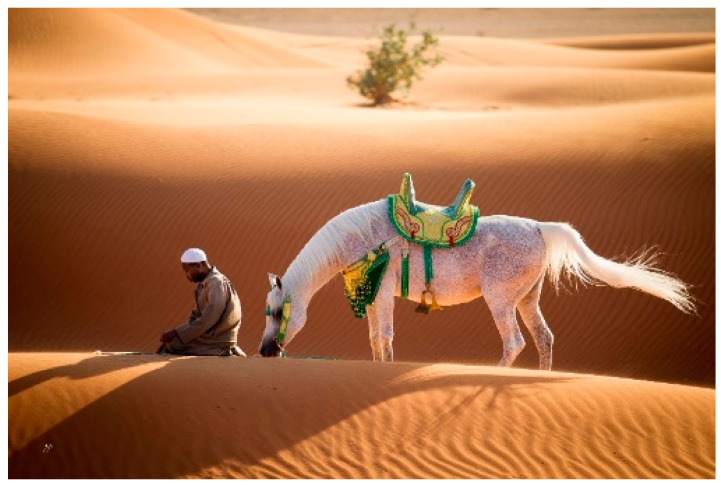
Arab horse in the desert (photo credit: Glenn Jacobs).

**Figure 2:**
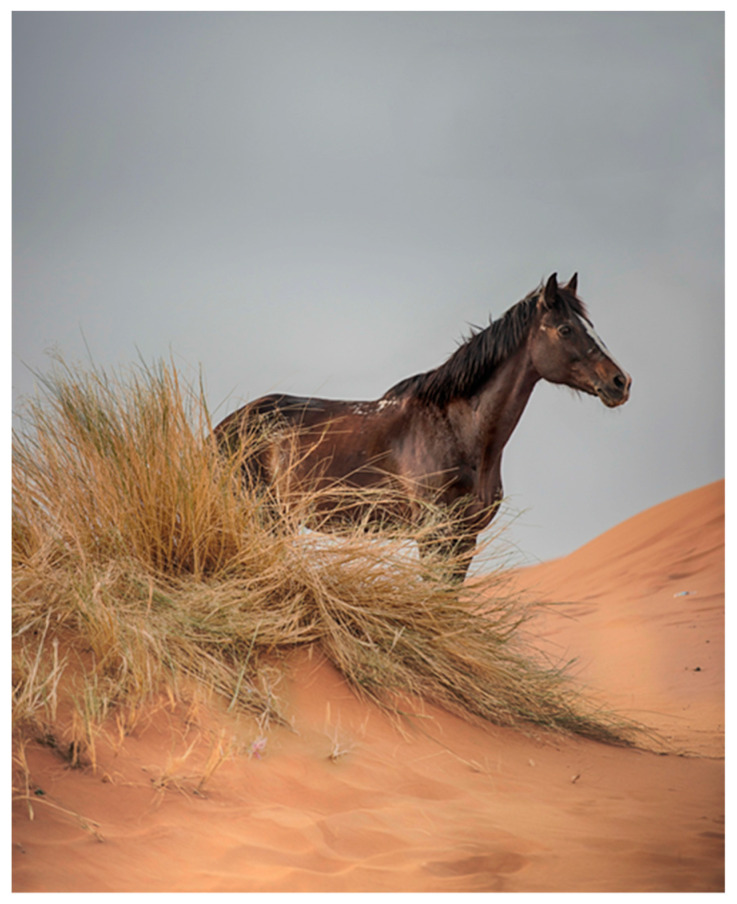
Arab-Barb horse (photo credit: Paula Da Silva).

**Figure 3:**
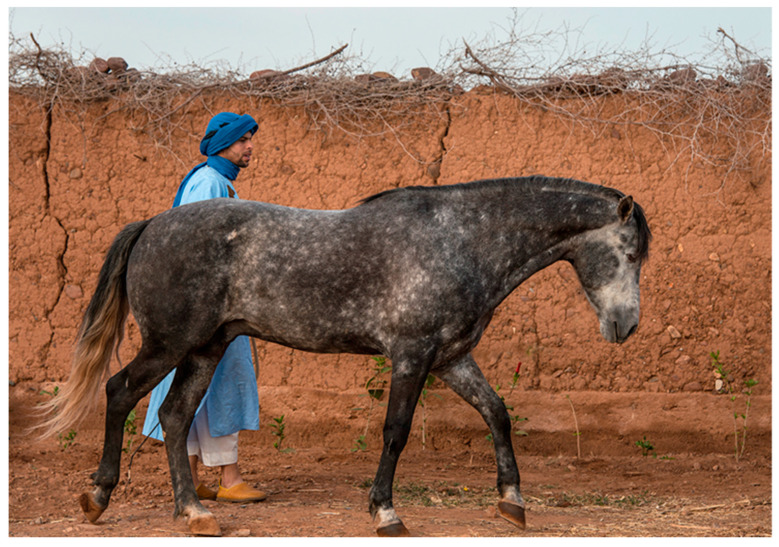
Grey Barb stallion (photo credit: Paula Da Silva).

Arabian (Figure 1), Barb (Figure 2) and Arab-Barb (Figure 3) horses are the original breeds in the MENA region. The three breeds have been bred geographically close for hundreds of years. The Arabian and Barb horses hold a prominent place in the MENA region and are recognised as the noblest and most influential breeds due to their contribution in the development of many other horse breeds worldwide. A recent analysis of Y chromosome diversity revealed that Barb horses cluster with barouqe, sorraia, and Spanish breeds [5]. Barbs were bred with Arabian horses resulting in the foundation of Arab-Barb horses, known for their high economic value due to their stamina and courage, maintenance and efficient feeding [6,7].

The Arabian, Barb and Arab-Barb horses are selectively bred to have the preferred traits that improve the phenotypic features or the aesthetic appeal and enhance athletic performance. Considering the origin of Arabian and Barb horses and their great influence on other breeds worldwide, this study aimed at screening a cohort of Arabian, Barb, Arab-Barb horses for the three genetic disorders as the Cerebellar Abiotrophy (CA), Lavender Foal Syndrome (LFS) and Severe Combined Immunodeficiency (SCID), in MENA horse populations. The obtained information can shed new light on the carriers of the studied diseases.

## 2. Materials and Methods

The Scientific Council approved the research of the Faculty of Nature and Life Sciences (Report of Faculty Scientific Council #05 dated 11 November 2020), University of Bejaia, Algeria). Concerning the ethical aspects, the experimental procedure was performed according to good veterinary practice under farm conditions.

### 2.1. Sampling

A total of 177 horses were chosen randomly and investigated for each of the three genetic disorders. Follicle hair samples from purebred Arabian (*n* = 80), Barb (*n =* 41) and Arab-Barb horses (*n =* 56) were used in this study. Thirty hairs 7 cm long were pulled out at the root of the neck or tail skin. The samples collected were preserved in an individually labelled paper envelope stored at room temperature until use for genetic disorders screen. Horses were checked clinically by a veterinarian. The neurologic examination evaluates (1) the cranial nerves, (2) the gait, or walk, (3) the neck and front legs, and (4) the torso, hind legs, anus, and tail. None of the horse showed signs of lowering reflexes of the head deterioration, constant pacing, seizures, a head turn or circling in one direction or other unusual head movements. None of the horses showed any signs of dysfunction within gait evaluation like circling, weakness or complete paralysis of any limbs, falling, stumbling, rolling, or loss of coordination. For evaluation of neck and front legs there were no evidence of pain, loss of muscle tone or cramp in the neck. None of the horses showed signs of loss of feeling or hypersensitivity to light touch or pinpricking, and loss of muscle mass. Therefore, the veterinarian claims all animals involved in study as healthy. Without suspicion of disease, there was no reason to order blood tests.

### 2.2. DNA Isolation and Genotyping

Genomic DNA was extracted from all samples using the Sherlock AX kit (A&A Biotechnology, Gdańsk, Poland) according to protocol. The DNA quality and concentration were measured with NanoDrop 2000 spectrophotometer (Thermo Fisher Scientific, Waltham, MA, USA).

#### 2.2.1. Cerebellar Abiotrophy (CA)

The PCR-ACRS method was designed to identify the single nucleotide polymorphism that determined the CA occurrence (rs397160943, NC_009145.3:g.13122415C>T; ENSECAT 00000024892.2:c.284G>A, ENSECAP00000020698.1:p.Arg95His). The primers were designed using Primer3 Input (version 0.4.0) and *TOE1* gene (ENSECAG00000023204) reference (Table 1), and due to the modified primer sequence, the artificial restriction site for *HpyCH4*III (BioLabs, New England, Ipswich, MA, USA) was created. The PCR was obtained using 2xPhanta Max Master Mix (Vazyme, Polgen, Łódź, Poland), and after 16 h digestion, the products were obtained as follows: C allele-111, 22 bp and T allele-133bp.

#### 2.2.2. Severe Combined Immunodeficiency (SCID)

The five base pair deletion (TCTCA) in *PRKDC* gene was detected using Sanger sequencing. The primers spine mutation site was designed using Primer3 Input (version 0.4.0) based on ENSECAG00000020168 reference (Table 1). The PCR product was obtained using Ampli Tag 360 DNA Polymerase (Applied Biosystems, Thermo Fisher Scientific, Waltham, MA, USA) according to protocol, and purified using EPPIC (A&A Biotechnology, Gdańsk, Poland). Next sequencing was performed with BigDye Terminator v3.1 Cycle Sequencing Kit (Applied Biosystems, Thermo Fisher Scientific, Waltham, MA, USA), Big Dye Xterminator Purification Kit (Applied Biosystems, Thermo Fisher Scientific, Waltham, MA, USA) and 3500XL Genetic Analyser platform (Applied Biosystems, Thermo Fisher Scientific, Waltham, MA, USA).

#### 2.2.3. Lavender Foal Syndrome (LFS)

The LFS genetic background mutation (*MYO5A* gene; g.138235715del) was detected using the Sanger sequencing method according to the same procedure described for SCID disorder. The primers used for *MYO5A* gene were designed based on ENSECAG00000021742 reference (Table 1).

For all three disorders, the negative and positive samples (heterozygote horses for each disorder) were included in every analysis.

## 3. Results and Discussion

Genetic disorders can have a dramatic impact on horse’s breeding. These disorders are mainly responsible for foal losses and significant economic losses due to the treatments and maintenance costs during pregnancy and breeding. Cerebellar Abiotrophy (CA), is a progressive neurological disease characterised by the degeneration of cerebellar Purkinje cells, and it is likely caused by an intrinsic metabolic disorder [8], which affects many animal species [9]. It has been demonstrated that CA is recognised almost exclusively in the Arabian horse breed [10]. However, the probability of CA mutation occurrence is present in other Arabian-cross horse breeds [11]. CA clinical symptoms usually are developed dramatically between the age of 6 weeks and 4 months, represented mainly by ataxia, hypermetria, intention head tremors, and the absence of a menace response. CA has an autosomal recessive mode of inheritance. The genetic mutation responsible for the disorder would be a single nucleotide polymorphism (SNP) located in exon 4 of *TOE1* gene (Target Of Early Growth Response 1) on equine chromosome 2, resulting in the incorporation of arginine instead of histidine at this position [12]. Therefore, crossing two carriers results in 25% affected foals in the population [12]. Reveal the number of horses in each genotype group and the percentage of each genotype/allele, 2.8% of the investigated horses (5 Arabian horses) were heterozygous for the rs397160943 SNP in *TOE1* gene at ECA2 with allele frequency of 0.1%, and no homozygous-affected horses for the mutation were identified (Table 2).

The CA is commonly recognised in Arabian horses, and studies showed that other breeds are at risk for the disease if Arabian founders were used as breeding stock [11]. The CA appears to be transmitted with a low frequency to other breeds descended from Arabian founders like the six heterozygous horses reported by Brault et al. [12], comprising 2 Trakehners, one Welsh pony, and 3 Bashkir Curly horses. Recent studies investigated the carrier’s frequency in 808 purebred Arabian horses, and its results confirmed that CA-related mutations tend to show a high allele frequency in Arabian horses [13].

Lavender Foal Syndrome (LFS) is a rare autosomal recessive lethal genetic coat colour-associated disorder combined with severe neurological symptoms. Arabian horse with heterozygous genes represents a carrier case, while that the homozygous genes individuals are mostly dying within a few hours or days [14]. Regarding the Lavender Foal Syndrome (LFS), our investigated horses were clear of this lethal disorder. Results recently obtained by Bugno-Poniewierska et al. [13] confirmed the absence of LFS in the studied group of purebred Arabian horses in Poland, indicating that the lethal allele was not introduced yet in some populations. To date, reports from South Africa, Croatia, and Egypt reported the incidence of LFS carriers [15,16,17]. Therefore, the occurrence of mutated alleles in horse population from the MENA regions is not inconceivable.

Severe Combined Immunodeficiency (SCID) is an autosomal recessive genetic disease, first described in two Arabian foals, full siblings 70 years ago [18]. Later studies confirmed that Equine SCID primarily affects Arabian horses and their crossbreeds [19]. SCID is characterized mainly by the inability of foals to produce antigen-specific immune responses due to a lack of functional B and T lymphocytes. The genetic basis of Equine SCID is the deletion of 5 base pairs (TCTCA) in the DNA-protein kinase catalytic subunit (DNA-PKcs) located in the short arm of chromosome 9 (ECA9p12) [20,21]. The obtained results confirmed the presence of the five nucleotides deletion within *PRKDC* associated with SCID disorder in the studied horses. The data shows that 5.7% of the studied horses (10 horses) are SCID carriers with a total SCID-associated allele frequency of 0.03. The ten carriers were nine Arabian and one Arab-Barb horses (Table 3).

In other investigated horse populations, researchers determined the frequency of SCID among 21 Arabian and Arabian crossbred horses in Morocco, where SCID carriers were representing 14 (7%) Arabian horses and 6 (4%) Arab-Barb horses [22]. While in the genetic screening of Arabian horses in Turkey, no SCID carrier cases were identified [23]. Moreover, a recent report confirmed introducing SCID-associated allele in three heterozygous Polish Arabian horses with a low frequency that did not exceed the threshold of 0.004 [13].

## 4. Conclusions

This study allowed us to identify the alleles responsible for both equine genetic diseases CA and SCID among Arabian horses in the MENA region. Investigated genetic disorders are autosomal, recessive, hereditary diseases occurring in the horse populations, and according to literature, a carrier of the disease-causing allele shows no clinical symptoms. With the arising of revolutionary research technologies and investigating methods, breeders became more aware of the best procedures to enhance their production and minimise the risks of genetic disorders that might negatively affect their profits. Therefore, our study strongly supports the need for genetic testing in Arabian and Arabian-crossed horses in the MENA region.

## Figures and Tables

**Table 1:** The primers used for detection of each polymorphism.

Gene	Primers	Method Used for Genotyping
*TOE1*	F: GGATCTCAACCCTCCTCTCC R: CGTGTGTCATGCTGCCAGGAaCC	PCR-ACRS
*PRKDC*	F: GGTAGCTTTGTGTTCCTGTTG R: TTCTCTCATTGCCAGAAGCA	Sanger sequencing
*MYO5A*	F: CAGAGCCTGAAGGAGGAGAA R: GTCAGCCGGGTGATCTCAT	Sanger sequencing

**Table 2:** The genotypes and alleles frequency distribution of rs397160943 SNP in *TOE1* gene related with CA (Cerebellar Abiotrophy) occurrence.

	Genotypes (N/%)			Alleles	
Breed	CC	CT	TT	C	T
Barb	41	-	-	1	
	100%				
Arab Barb	56	-	-	1	
	100%				
Arabian	75	5	-	0.94	0.06
	93.7%	6.3%			
Total	172	5	-	0.99	0.01
	97.2%	2.8%			

The data presents the numbers of horses in each genotype group and the percentage of each genotype/allele; N—number of horses detected; ‘-‘—the lack of detected animals with given genotype; C—the reference allele; T—allele related with Cerebellar Abiotrophy disorder.

**Table 3:** The genotypes and alleles distribution of deletion within *PRKDC* gene related with SCID occurrence in different horse breeds.

	Genotypes (N/%)			Alleles	
Breed	WT/WT	WT/SCID	SCID/SCID	WT	SCID
Barb	41	-	-	1	
	100%				
Arab Barb	55	1	-	0.99	0.01
	98.2%	1.3%			
Arabian	71	9	-	0.94	0.06
	88.7%	11.3%			
Total	167	10	-	0.97	0.03
	94.3%	5.7%			

The data presents the numbers of horses in each genotype group and the percentage of each genotype/allele; N—number of horses detected; ‘-‘—the lack of detected animals with given genotype; WT—wild allele (reference); SCID—the deletion of TCTCA motif within *PRKDC*.

## Data Availability

The data presented in this study are available on request from the corresponding author. The data are not publicly available due to fact that the horse owners asked for discretion and not to disclose the results to the public.
